# Prevalence and Molecular Epidemiology of Intestinal Colonization by Multidrug-Resistant Bacteria among Hematopoietic Stem-Cell Transplantation Recipients: A Bulgarian Single-Center Study

**DOI:** 10.3390/antibiotics13100920

**Published:** 2024-09-26

**Authors:** Denis Niyazi, Stoyan Vergiev, Rumyana Markovska, Temenuga Stoeva

**Affiliations:** 1 Clinical Microbiology Laboratory, University Hospital “St. Marina”—Varna, 9010 Varna, Bulgaria; temenuga.stoeva@abv.bg; 2 Department of Microbiology and Virology, Medical University—Varna, 9002 Varna, Bulgaria; 3 Department of Ecology and Environmental Protection, Technical University of Varna, 9010 Varna, Bulgaria; stvergiev@gmail.com; 4 Department of Medical Microbiology, Medical University—Sofia, 1431 Sofia, Bulgaria; markovska73@abv.bg

**Keywords:** fecal colonization, multidrug-resistant bacteria, hematopoietic stem-cell transplantation

## Abstract

**Background/Objectives**: Intestinal colonization by multidrug-resistant (MDR) bacteria is considered one of the main risk factors for invasive infections in the hematopoietic stem-cell transplant (HSCT) setting, associated with hard-to-eradicate microorganisms. The aim of this study was to assess the rate of intestinal colonization by MDR bacteria and their microbial spectrum in a group of post-HSCT patients to study the genetic determinants of beta-lactam and glycopeptide resistance in the recovered isolates, as well as to determine the epidemiological relation between them. **Methods**: The intestinal colonization status of 74 patients admitted to the transplantation center of University Hospital “St. Marina”—Varna in the period January 2019 to December 2021 was investigated. Stool samples/rectal swabs were screened for third-generation cephalosporin and/or carbapenem-resistant Gram-negative bacteria, methicillin-resistant *Staphylococcus aureus* (MRSA), vancomycin-resistant enterococci (VRE), and *Stenotrophomonas maltophilia*. Identification and antimicrobial susceptibility testing were performed by Phoenix (BD, Sparks, MD, USA) and MALDI Biotyper sirius (Bruker, Bremen, Germany). Molecular genetic methods (PCR, DNA sequencing) were used to study the mechanisms of beta-lactam and glycopeptide resistance in the collected isolates, as well as the epidemiological relationship between them. **Results**: A total of 28 patients (37.8%) were detected with intestinal colonization by MDR bacteria. Forty-eight non-duplicate MDR bacteria were isolated from their stool samples. Amongst them, the Gram-negative bacteria prevailed (68.8%), dominated by ESBL-producing *Escherichia coli* (30.3%), and followed by carbapenem-resistant *Pseudomonas* sp. (24.2%). The Gram-positive bacteria were represented exclusively by *Enterococcus faecium* (31.2%). The main beta-lactam resistance mechanisms were associated with CTX-M and VIM production. *VanA* was detected in all vancomycin-resistant enterococci. A clonal relationship was observed among *Enterobacter cloacae* complex and among *E. faecium* isolates. **Conclusions**: To the best of our knowledge, this is the first Bulgarian study that presents detailed information about the prevalence, resistance genetic determinants, and molecular epidemiology of MDR gut-colonizing bacteria in HSCT patients.

## 1. Introduction

The hematopoietic stem-cell transplant (HSCT) is an established therapeutic procedure since the 1950s, recommended for hematological malignancies (HMs) as well as for autoimmune diseases, neurological complications, and even solid tumors [1]. According to the Worldwide Network of Blood and Marrow Transplantation, there are more than 1700 transplant centers worldwide, and more than 90,000 HSCTs are performed each year [2]. Despite the tremendous benefit of this therapeutic procedure, adverse effects due to prolonged immunosuppression, mucositis, graft-versus-host disease, etc., are frequently observed. Bloodstream infections (BSIs) are among the most severe infectious complications after HSCT, with an incidence ranging between 11% and 40% [3,4]. The development of BSIs depends on many factors, but intestinal colonization by multidrug-resistant (MDR) bacteria is among the most well-documented risk factors for related BSIs [5,6]. Chemotherapy, ablative conditioning regimen, and graft-versus-host disease are associated with mucosal barrier injury, resulting in microbial translocation into the bloodstream [7]. It has also been reported that the BSI rate is significantly higher in colonized than in non-colonized patients (50% vs. 7.5%) [8]. Taur et al. demonstrated in their study that HSCT patients with MDR fecal colonization are five to nine times more likely to have a BSI episode caused by the same strain compared to those without colonization [9].

The aim of this study was to assess the rate of intestinal colonization by MDR bacteria and their microbial spectrum in a group of post-HSCT patients and to study the genetic determinants of beta-lactam and glycopeptide resistance in the recovered isolates, as well as to determine the epidemiological relation between them.

## 2. Results

**Figure 1:**
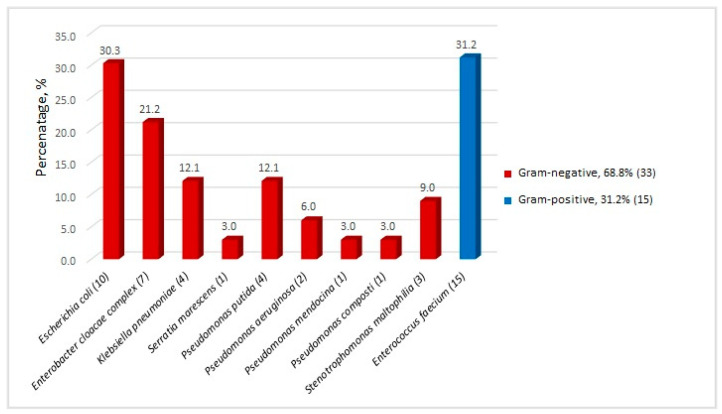
MDR bacterial isolates recovered from stool samples of 28 post-HSCT patients, % (*n*).

A total of 48 MDR microbial isolates were obtained from the stool samples of 28 of 74 screened patients (37.8%) included in this study. The species diversity of the colonizing agents is presented in Figure 1.

The antimicrobial resistance rates in all Gram-negative bacterial isolates are presented in Table 1.

In this study, a total of 15 (31.2%) vancomycin-resistant *E. faecium* (VREfm) (MIC > 16 µg/mL) isolates were detected. They were also resistant to teicoplanin, all beta-lactams, aminoglycosides, and quinolones but remained susceptible to linezolid.

All 48 MDR fecal isolates were studied to identify the genetic mechanisms of their beta-lactam and glycopeptide resistance. In 22 *Enterobacterales* isolates (45.8%) resistant to third-generation cephalosporins, the following genes were found: *bla*_CTX-M_ (*n* = 20; 90.9%; *E. coli*, *n* = 9; *K. pneumoniae*, *n* = 3; *E. cloacae* complex, *n* = 7; *S. marcescens*, *n* = 1), *bla*_TEM_ (*n* = 14; 63.6%; *E. coli*, *n* = 5; *K. pneumoniae*, *n* = 2; *E. cloacae* complex, *n* = 6; *S. marcescens*, *n* = 1), and *bla*_SHV_ (*n* = 4; 18.2%; *K. pneumoniae*, *n* = 4). Different combinations of *bla* genes were found in fourteen isolates (63.6%): *bla*_CTX-M_ + *bla*_TEM_ (*E. coli*, *n* = 4; *K. pneumoniae*, *n* = 2; *E. cloacae* complex, *n* = 6; *S. marcescens*, *n* = 1); *bla*_CTX-M_ + *bla*_SHV_ (*K. pneumoniae*, *n* = 3), and *bla*_CTX-M_ + *bla*_TEM_ + *bla*_SHV_ (*n* = 2, *K. pneumoniae*).

Among nine (18.8%) carbapenem-resistant (CR) isolates (*P. putida*, *n* = 4; *P. aeruginosa*, *n* = 2; *P. mendocina*, *n* = 1; *P. composti*, *n* = 1; *E. cloacae* complex, *n* = 1), seven isolates carried *bla*_VIM_ (77.8%). Two CR isolates (*P. aeruginosa* and *P. putida*) did not yield amplification products with any of the used primer sets, suggesting other mechanisms involved (Table 2).

The DNA sequencing confirmed the presence of *bla*_CTX-M-15_ (*n* = 5), *bla*_CTX-M-3_ (*n* = 3), *bla*_TEM-1_ (*n* = 1), *bla*_SHV-1_ (*n* = 1), *bla*_SHV-12_ (*n* = 1), *bla*_VIM-1_ (*n* = 1), and *bla*_VIM-2_ (*n* = 6) in 16 representative isolates, selected on the basis of their antimicrobial resistance phenotype/genotype and epidemiological typing results (Table 2).

*VanA* was identified as the mechanism responsible for glycopeptide resistance in all vancomycin-resistant *E. faecium*.

A total of 36 isolates were studied for their genetic relatedness: *E. coli*, *n* = 10; *E. cloacae* complex, *n* = 7; *K. pneumoniae*, *n* = 4; and *E. faecium*, *n* = 15.

Among *E. coli* isolates (*n* = 10), eight ERIC types were identified, six of which were unique. Types a and b are represented by two isolates each.

**Figure 2:**
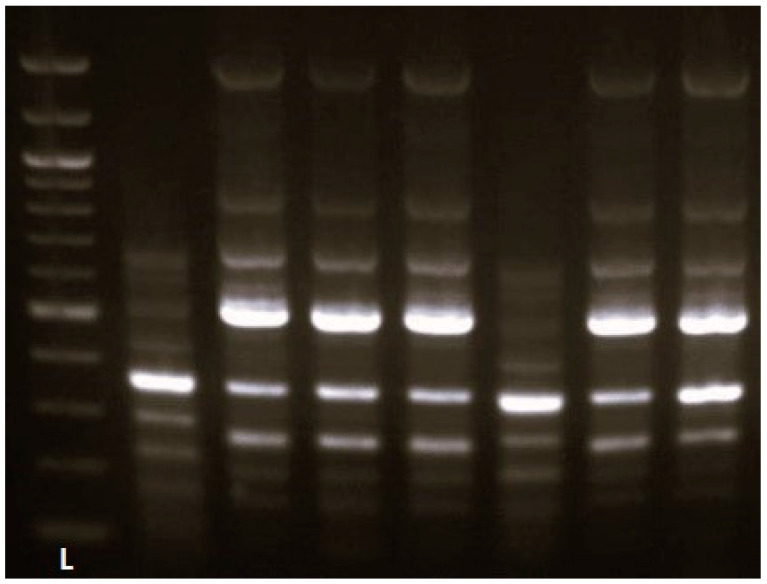
Gel electrophoresis image of *E. cloacae* complex typed by ERIC PCR. L—100 bp DNA Ladder.

In the *E. cloacae* complex group (*n* = 7), two ERIC types (A, B) were found, of which type A was dominant, represented by five isolates (Figure 2).

All *K. pneumoniae* isolates exhibited different ERIC profiles (Table 2).

In the *E. faecium* group (*n* = 15), six RAPD types were identified; Type A prevailed (46.7%). RAPD types B and C included three and two isolates, respectively. Unique RAPD profiles were found in three isolates.

In the current study, we did not detect colonizing agents such as methicillin-resistant *Staphylococcus aureus* (MRSA), CR *Acinetobacter baumannii,* or other CR Gram-negative bacteria.

## 3. Discussion

Patients with hematological malignancies are at high risk for infectious complications because of chemotherapy and immunosuppression due to the underlying disease. Infections caused by MDR pathogens result in increased mortality compared to those caused by bacteria with preserved susceptibility [10]. Nowadays, infectious complications associated with MDR organisms in this patient population have been increasingly reported [11]. One of the major risk factors for invasive infections caused by MDR microorganisms is prior colonization by resistant bacteria, a finding reported by many authors and documented in our previous study [12,13,14]. Because a large proportion of HM patients are on long-term antimicrobial therapy, those undergoing HSCT are at increased risk of colonization by MDR bacteria prior to transplantation [15].

Several recent studies have demonstrated the role of the gut microbiota in HSCT for the development of BSIs [16,17,18,19]. The transplantation procedure negatively affects the intestinal microbiota. The human gut microbiota interacts with the host immune system, and reduced microbial diversity results in an inadequate immune response [20]. In patients with suppressed immune systems following HSCT and after antibiotic exposure, altered gut microflora may affect immune recovery and can explain the increased death rate associated with an infectious cause in colonized individuals [15].

To observe the colonization status of the patients included in this study, fecal screening for MDR bacteria was performed. We found a high pre-transplant MDR colonization rate (37.8%) among the tested patients. Similar results were reported by Bilinski et al., who identified 31% of their HSCT patients with intestinal carriage of organisms exhibiting multidrug resistance [16]. Other authors who also monitored the colonization status of patients after allogeneic HSCT found a much higher prevalence of fecal colonization (53.8%) [15].

Among all bacterial isolates, Gram-negative bacteria were found to dominate (68.8%). The representatives of order *Enterobacterales* were the most abundant (66.7%), with *E. coli* being the most common isolate. Gram-positive bacteria were solely represented by *E. faecium* (31.2%). Our results differ notably from those of Scheich and Bilinski, who reported a higher incidence of fecal colonization by Gram-positive bacteria (85.9%), mainly *E. faecium* (55%) [15,16].

Twenty-two Gram-negative isolates (45.8%) were identified as extended-spectrum beta-lactamase (ESBL) producers. In contrast to this result, in a three-year Polish study following 107 patients after allogeneic HSCT, 20% intestinal colonization with ESBL producers was reported [16]. Similarly, another study from 2006 to 2016 in Germany among patients with acute myeloid leukemia and subsequent allo-HSCT found 20.4% intestinal carriage of ESBL-producing *Enterobacteriaceae* [15]. Genes coding for ESBL production have a global distribution and are more and more frequently detected in various Gram-negative bacteria [21]. Among them, *bla*_CTX-M_ is the most prevalent, a result also confirmed by the findings in the current study (90.9%) [22,23]. Fecal colonization by ESBL producers is known to be an independent risk factor for invasive infections caused by the colonizing strain in the neutropenic stage of the HSCT [24]. In addition, the main factors leading to microbial colonization with *bla*-harboring bacteria are previous beta-lactam exposure, older age, and coexisting chronic conditions [25].

Among the ESBL-producing bacteria in our study, reduced susceptibility to other antibiotics like aminoglycosides, sulfonamides, and fluoroquinolones was documented. Colonization with bacteria, demonstrating significantly reduced susceptibility to trimethoprim/sulfamethoxazole, ciprofloxacin, and piperacillin/tazobactam (resistance above 40%), is alarming, as these agents are often preferred for prophylaxis and empiric therapy in individuals with febrile neutropenia. Amongst the aminoglycosides, in contrast to gentamicin, amikacin was the less-affected agent and had generally preserved activity (resistance below 5%). Opposite to our results, a study by Scheich et al. reported a complete lack of susceptibility to fluoroquinolones in all tested ESBL producers, an event probably caused by the gene combination accountable for fluoroquinolone and beta-lactam resistance in the same plasmid [15].

Of all gut-colonizing bacteria, the prevalence of CR Gram-negative bacteria was 18.8%, mainly represented by CR isolates of *Pseudomonas* sp. (88.9%). Amongst the CR isolates, eight exhibited resistance to ceftazidime/avibactam, and all were colistin-susceptible. The carbapenem resistance was mainly associated with VIM-2 (in four isolates of *Pseudomonas* sp.) and, to a lesser extent, with VIM-1; the latter was found in one *E. cloacae* complex isolate. Sporadic cases of *P. aeruginosa* carrying VIM metallo-carbapenemases have been described in Bulgaria, with the first report in 2006 [26]. Recently, Strateva et al. described a *bla*_VIM-2_-harboring *P. aeruginosa* belonging to the high-risk *ST111* group [27]. Similarly, *E. cloacae complex* carrying *bla*_VIM-1_ have been detected in different parts of the world and were mainly associated with hospital clonal spread [28,29].

In contrast to our findings, a lower CR Gram-negative colonization rate among HSCT recipients was reported by Scheich et al. (8.5%), with a predominance of *P. aeruginosa* [15]. A similar rate was reported by Bilinski et al. (6%) [16]. Giannella et al. screened patients after solid organ transplantation for 8 years and documented a prevalence of 26.6% intestinal CR *Enterobacteriaceae* (CRE) colonization [19]. In a meta-analysis by Luo et al. on Gram-negative gut-colonizers in patients with HM, the reported pooled CRE rate was 21.7%, which correlates with our findings (18.8%) [30]. The CRE rate is lower than ours in Europe (8.9%), the Eastern Mediterranean (14.9%), and the Americas (15.5%), but it is much higher in Southeast Asia (57.4%) [30].

In Bulgaria, between 2017 and 2018, Stankova et al. carried out a research study on MDR fecal colonization among patients hospitalized in six large hospitals across the country, comparing the results to those obtained from healthy individuals. A higher rate of colonizing MDR bacteria (ESBL and carbapenemase-producers) in the hospitalized group of patients (10%) compared to that in the healthy individuals (1%) was reported [31]. The in-hospital clonal spread of MDR bacteria and the high antibiotic selective pressure were reported as possible factors influencing this difference [31]. Regarding HSCT, the major risk factors for CRE intestinal colonization in this patient population are related to both patients and treatment factors. Among these, prophylactic antimicrobial usage (especially fluoroquinolones), history of prior exposure to antimicrobial agents, combined use of antibiotics, and carbapenem usage are proven statistically significant risk factors for CRE colonization [32,33]. In addition, prolonged neutropenia, prior and prolonged hospitalization and/or multiple hospital stays, transfer between units, ICU admission, and the presence of foreign bodies are also involved [32,33]. Not in last place, poor patient hygiene and/or inadequate patient isolation significantly contribute to the hospital spread of the pathogen [32].

*Stenotrophomonas maltophilia* was long considered a saprophytic microorganism and harmless colonizer in the hospital environment, but its role as a pathogen that can give rise to severe complications in the immunosuppressed host is increasingly recognized [34]. Due to its inherited chromosomal resistance, the therapeutic options are often limited [35]. Furthermore, colonization by *S. maltophilia* can be an indicator of a long hospital stay and broad-spectrum antibiotic overconsumption [36]. In our study, three MDR isolates of *S. maltophilia* were identified, all demonstrating resistance to trimethoprim/sulfamethoxazole, with preserved susceptibility only to colistin.

In this study, all isolated Gram-positive bacteria were identified as VREfm, also exhibiting resistance to teicoplanin. The VRE colonization rate was relatively high (31.2%). Similar results were reported in a Polish study conducted between 2010 and 2013 among HSCT recipients—21% [16]. A notably higher percentage of VRE was reported in a German study, encompassing more than a 10-year period amongst transplant recipients—85.9% [15]. The variation in the prevalence of VRE colonization among the different studies may be explained by differences in the underlying disease, the treatment protocol prior to transplantation, and the use of antibiotics for prophylaxis, such as fluoroquinolones, known for being a risk factor for VRE colonization [15]. The intestinal VRE carriage is associated with decreased bacterial diversity and a higher rate of graft-versus-host disease and its often life-threatening course [15]. As was demonstrated in the present study, the spectrum of Gram-positive MDR bacteria associated with intestinal colonization was dominated solely by VREfm isolates, all positive for the *vanA* gene. Between 2017 and 2018, Hitkova et al. screened hospitalized patients for fecal carriage of glycopeptide-resistant enterococci and reported a prevalence of 29.4% VRE (*E. faecium, E. casseliflavus,* and *E. gallinarum*) and the resistance was mainly mediated by the *vanC* gene. The authors identified *vanA* in only 5.5% [37]. Consistent with our results, in a study conducted by Strateva et al. involving isolates of *E. faecium* from three large Bulgarian hospitals, all 51 VRE isolates were *vanA*-positive [38].

In recent years, MDR bacteria have been transformed into a major problem in the healthcare system worldwide, and healthcare institutions continuously face outbreaks associated with such pathogens [39]. According to EARS-NET data for 2020, the incidence of invasive infections caused by resistant organisms has steadily increased during recent years [40,41]. The problematic and difficult-to-treat MDR pathogens, such as ESBL and carbapenemase-producing Gram-negative bacteria, VRE, and MRSA, develop another pathogenicity factor—the ability to disseminate clonally in the hospital environment, causing nosocomial outbreaks and endemic situations.

In the present study, among the 10 fecal *E. coli* isolates, a genetic relationship was only demonstrated between isolates, which formed small groups of two representatives, all carrying the *bla*_CTX-M_ gene. Results like ours were reported in a study by Kharrat et al. encompassing a 10-year period. The authors studied antimicrobial resistance and the epidemiological association between bacterial isolates obtained from patients undergoing HSCT. They reported an increased prevalence of third-generation cephalosporin-resistant *E. coli* (43%) carrying *bla*_CTX-M_. The authors found a high genetic diversity of five pulsotypes, each represented by two isolates [41]. Similar results were obtained by Uemura et al., who studied *E. coli* isolates obtained from hematology patients. A non-clonal prevalence associated with *bla*_CTX-M_, *bla*_SHV,_ and *bla*_TEM_ genes was reported [42]. On the contrary, in a Bulgarian study conducted by Markovska et al. on ESBL-producing *Enterobacteriaceae* from fecal samples, a clonal relation between most of the isolates was reported, and *bla*_CTX-M_ was also identified as the main resistance determinant [43].

*E. cloacae* complex is mainly known for being an opportunistic pathogen accountable for a wide range of hospital-acquired infections [44,45,46]. In the current study, among the *E. cloacae* complex isolates recovered from seven different patients, one dominant ERIC A type was identified, represented by five identical strains carrying the *bla*_CTX-M_ gene, one of which was also *bla*_VIM-1_-positive and carbapenem-resistant. Our results confirm the potential of *E. cloacae* complex to acquire different resistance determinants and to spread clonally. These results also confirm the findings of Dimitrova et al. for the clonal spread of CTX-M-15-producing *E. cloacae* complex among patients admitted to the Clinical Hematology ward of St. Marina University Hospital—Varna during the 2014–2017 period [47]. Similarly, during the COVID-19 pandemic, Mulllié et al. investigated a hospital outbreak associated with *E. cloacae* complex in an ICU in a French university hospital and found all environmental and clinical isolates (including from fecal samples) clonally related and *bla*_VIM-4_-positive [39].

Amongst the members of the order *Enterobacterales*, *K. pneumoniae* is the most problematic pathogen associated with extremely difficult-to-treat infections because of its MDR and even pan-drug resistance [48]. The ability to easily acquire multiple resistance determinants and the presence of various other virulence factors make this organism a superbug [49]. These specificities also amplify the potential of *K. pneumoniae* to colonize patients hospitalized for long periods in medical institutions [50]. In the current study, a low incidence of third-generation cephalosporin-resistant *K. pneumoniae* isolates with no genetic similarity was found. Kharrat et al. reported similar data. For a 10-year period, the authors identified 17 unique pulsotypes among 19 ESBL-producing *K. pneumoniae* isolated from fecal and other clinical samples of HSCT patients [41]. On the contrary, researchers from Japan demonstrated the potential of *K. pneumoniae* to spread clonally in the hospital setting by identifying a cluster of closely related strains causing a hospital outbreak, which affected patients from several wards (including Hematology) [42].

Regarding the increasing global prevalence of MDR bacteria, the incidence of VRE is also on the rise [51]. Similar to MDR Gram-negative bacteria, VRE are associated with difficult-to-treat infections as well as with hard-to-control nosocomial outbreaks, becoming a serious medical challenge in many hospital settings [52]. In this study, the epidemiological relationship between 15 fecal isolates of VREfm was studied. One dominant RAPD A type represented by seven strains obtained from seven patients was identified, a result clearly demonstrating the potential of these bacteria for clonal spreading. As was mentioned, the vancomycin resistance in all isolates was *vanA*-associated. Between 2012 and 2013, 9454 fecal samples were tested for VRE carriage in Norway during a nosocomial outbreak in a surgical ward, and two clusters represented by strains of *E. faecium*, all *vanA* carriers, were identified [53]. Similarly, Weterings et al. also investigated a hospital outbreak caused by VREfm and found intestinal VRE colonization in a total of 140 patients, some of them with related BSIs. The epidemiological typing confirmed the dissemination of one *vanA*-positive VRE clone [51]. In concordance with our results, the study of de Artola et al. from 2015 performed in a Spanish hematology clinic reported *E. faecium* intestinal colonization in 18.9% of 117 tested patients, and 9% of the colonized individuals developed related invasive infections. In addition, this study also identified a hospital outbreak associated with three closely related pulsotypes carrying the *vanA* gene [54]. Similar results for hospital clonal dissemination of *vanA*-carrying VREfm, as well as an endemic situation associated with *vanB*-positive *E. faecium* in an Australian hospital, were described by Hughes et al. [55].

In addition to the documented clonal dissemination of MDR bacteria among HSCT patients in this study, we also found non-clonally related unique MDR isolates. It is considered that the non-clonal or restricted spread of these problematic organisms is related to effective hygiene measures implemented from patient admission to discharge, resulting in minimizing bacterial transmission between transplanted individuals [41].

## 4. Materials and Methods

### 4.1. Patient Characteristics

A total of 74 patients who were admitted to the Hematopoietic Stem-Cell Transplantation Unit of the University Hospital “Saint Marina”, Varna, Bulgaria, and underwent HSCT between January 2019 and December 2021 were included in the study. HSCT was chosen as salvage therapy for neoplastic disease in 71 (96%) patients and in 3 for multiple sclerosis. Most patients were diagnosed with multiple myeloma (35.1%) and non-Hodgkin’s lymphoma (20.3%). Autologous HSCT was performed in 49 (66.2%) and allogeneic in 25 (33.8%) of the cases.

### 4.2. Fecal Screening

Fecal screening for MDR bacteria is implemented as a routine test for all patients undergoing HSCT in our transplantation center. The pre-transplant colonization surveillance includes screening for 3rd-generation cephalosporin and/or carbapenem-resistant Gram-negative bacteria, methicillin-resistant *Staphylococcus aureus* (MRSA), vancomycin-resistant enterococci (VRE), and *Stenotrophomonas maltophilia*. The obtained specimens are inoculated on MacConkey agar with 1 mg/L cefotaxime, CHROMagar^TM^ CPE (Franklin Lakes, NJ, USA), and blood agar (OXOID, Hampshire, UK) and incubated for 24 h at 37 °C. During the studied period, a total of 242 fecal samples collected from 74 HSCT patients were tested. All bacterial isolates that met the criterion of the aforementioned MDR pattern were included in the study.

### 4.3. Species Identification

Species identification was done by the automated system Phoenix M50 (BD, Franklin Lakes, NJ, USA) and confirmed by the MALDI Biotyper Sirius (Bruker, Bremen, Germany).

### 4.4. Antimicrobial Susceptibility Testing

Antimicrobial susceptibility testing was performed by the Phoenix M50 (Franklin Lakes, NJ, USA), and the results were interpreted according to the EUCAST 2019/2021 [56]. Susceptibility to colistin, vancomycin, and teicoplanin was determined by the commercial microdilution test MIKROLATEST (Erba Lachema, Brno, Czech Republic).

### 4.5. Molecular-Genetic Experiments for Detection of Genes Associated with Antimicrobial Resistance

Polymerase chain reaction (PCR) was used to detect genes encoding resistance to beta-lactam antibiotics in Gram-negative bacteria (*bla*_SHV_, *bla*_CTX-M_, *bla*_TEM_, *bla*_KPC_, *bla*_NDM_, *bla*_VIM_, *bla*_IMP_, and *bla*_OXA-48_) and to glycopeptides in the enterococcal isolates (*vanA*, *vanB*, and *vanD*), as previously described [57,58,59,60].

### 4.6. DNA Sequencing

Sequencing of PCR products (*bla*_SHV_, *bla*_CTX-M-1_ group, and *bla*_TEM_) was performed with primers binding outside the coding regions [57]. The nucleotide sequence was analyzed using Chromas Lite 2.01 (Technelysium Pty Ltd., Brisbane, Australia) and DNAMAN version 8.0 Software (Lynnon BioSoft, Vaudreuil-Dorion, QC, Canada). PCR with a combination of *bla*_VIM_-specific primers (VIM-F and VIM-R) and oligonucleotides binding to conserved regions of class I integrons 5CS and 3CS was carried out [26,61]. The amplicons were sequenced by the Sanger method using the BigDye^®^ Terminator v3.1 Cycle Sequencing Kit (Thermo Fisher Scientific Inc, Waltham, MA, USA) on an Applied Biosystems 3130xl Genetic Analyzer (Thermo Fisher Scientific Inc, Waltham, MA, USA).

### 4.7. Epidemiological Typing

ERIC PCR was used to determine the genetic relationship between the collected Gram-negative isolates (*E. coli*, *E. cloacae* complex, and *K. pneumoniae*), and RAPD PCR was used for *E. faecium* isolates [62,63]. The similarity was measured by the Jaccard coefficient and unweighted pair group method with arithmetic mean (UPGMA). Isolates with a similarity index ≥ 0.9 (ERIC PCR) or ≥ 0.8 (RAPD PCR) were considered a clonal group. All analyses were done on PAST 4 software (https://www.nhm.uio.no/english/research/resources/past/, accessed on 9 March 2024).

All oligonucleotide sequences and basic PCR conditions used in the study are presented in Table 3.

## 5. Conclusions

To the best of our knowledge, this is the first study from Bulgaria that reports detailed information about the prevalence, resistance genetic determinants, and molecular epidemiology of MDR intestinal-colonizing bacteria in HSCT patients. Our data show a high prevalence of intestinal MDR colonization in this high-risk population, with predominant colonization by ESBL-producing *Enterobacterales*.

A regular and continuous screening strategy for MDR bacteria colonizing the gastrointestinal tract of HSCT patients is an active approach to prevent the spread of these problematic bacteria among the susceptible population. Knowledge about their colonization status allows for the early initiation of targeted antibiotic treatment during febrile neutropenia episodes, thus reducing the incidence and progression of MDR-related infections and the associated mortality.

## Figures and Tables

**Table 1:** Antimicrobial resistance rates in 33 third-generation cephalosporin-resistant Gram-negative isolates associated with intestinal colonization in 28 post-HSCT patients, *n* (%).

Organism (*n*)	FEP	TZP	MEM	CIP	G	AK	TSM	COL
*E. coli* (10)	10 (100)	2 (20.0)	0 (0.0)	1 (10.0)	4 (40.0)	0 (0.0)	4 (40.0)	0 (0.0)
*E. cloacae* complex (7)	7 (100)	5 (71.4)	1 (14.3)	6 (85.7)	7 (100)	0 (0.0)	2 (28.6)	0 (0.0)
*K. pneumoniae* (4)	4 (100)	3 (75.0)	0 (0.0)	4 (100)	3 (75.0)	1 (25.0)	3 (75.0)	0 (0.0)
*S. marcescens* (1)	1 (100)	0 (0.0)	0 (0.0)	1 (100)	1 (100)	0 (0.0)	0 (0.0)	NA
*Pseudomonas* spp. (8)	8 (100)	8 (100)	8 (100)	7 (87.5)	5 (62.5)	3 (37.5)	NA	0 (0.0)
*S. maltophilia* (3)	NA	NA	NA	NA	NA	NA	3 (100)	0 (0.0)

FEP, cefepime; TZP, piperacillin/tazobactam; MEM, meropenem; CIP, ciprofloxacin; G, gentamicin; AK, amikacin; TSM, trimethoprim/sulfamethoxazole; COL, colistin; NA, not applicable; *n*—number.

**Table 2:** Summary of the fecal screening findings.

Patient ID	Fecal Screening		ERIC/RAPD Type
	Isolate	Resistance Profile and Genetic Determinants	
2	*E. faecium*	VRE (*vanA*)	RAPD Aa1
5	*E. faecium*	VRE (*vanA*)	RAPD C
	*K. pneumoniae*	ESBL (CTX-M-15, SHV-1, TEM)	Unique
6	*E. faecium*	VRE (*vanA*)	RAPD Aa1
9	*P. putida*	CR (VIM-2)	
11	*E. cloacae*	ESBL (CTX-M-15, TEM-1)	ERIC A
14	*E. faecium *	VRE (*vanA*)	Unique
	*E. coli *	ESBL (TEM)	Unique
	*P. putida*	CR **	
15	*E. coli*	ESBL (CTX-M)	ERIC a
16	*P. putida*	CR (VIM-2)	
18	*E. faecium *	VRE (*vanA*)	Unique
	*E. coli*	ESBL (CTX-M, TEM)	ERIC b
19	*E. cloacae*	ESBL (CTX-M-15, TEM-1)	ERIC A
20	*S. maltophilia *		
	*E. faecium *	VRE (*vanA*)	RAPD Bb
	*E. faecium *	VRE (*vanA*)	RAPD Ba
	*K. pneumoniae *	ESBL (CTX-M, TEM, SHV)	Unique
	*P. putida *	CR (VIM-2)	
	*E. coli*	ESBL (CTX-M-15, TEM)	ERIC b
22	*E. coli*	ESBL (CTX-M)	Unique
26	*E. cloacae*	ESBL + CR (CTX-M-15, TEM-1, VIM-1)	ERIC A
28	*P. mendocina*	CR (VIM-2)	
32	*E. coli*	ESBL (CTX-M)	ERIC a
34	*E. faecium*	VRE (*vanA*)	Unique
35	*E. faecium*	VRE (*vanA*)	RAPD Aa1
36	*S. maltophilia*		
37	*E. faecium *	VRE (*vanA*)	RAPD Aa2
	*S. maltophilia *		
	*P. composti*	CR (VIM-2)	
42	*K. pneumoniae*	ESBL (SHV-12)	Unique
45	*E. cloacae*	ESBL (CTX-M-15, TEM-1)	ERIC A
47	*E. faecium*	VRE (*vanA*)	RAPD Ab
48	*E. coli*	ESBL (CTX-M)	Unique
49	*P. aeruginosa *	CR **	
	*E. faecium *	VRE (*vanA*)	Unique
	*E. cloacae*	ESBL (CTX-M-15, TEM-1)	ERIC A
50	*P. aeruginosa *	CR (VIM-2)	
	*E. faecium*	VRE (*vanA*)	Unique
53	*E. faecium *	VRE (*vanA*)	RAPD Ab
	*E. coli*	ESBL (CTX-M)	Unique
60	*E. cloacae *	ESBL (CTX-M-3, TEM)	ERIC B
	*E. faecium *	VRE (*vanA*)	RAPD Aa1
	*E. coli *	ESBL (CTX-M-3, TEM)	Unique
	*K. pneumoniae *	ESBL (SHV, CTX-M)	Unique
	*E. coli*	ESBL (CTX-M-3)	Unique
67	*S. marcescens *	ESBL (CTX-M-15, TEM)	
	*E. cloacae*	ESBL (CTX-M-3, TEM)	ERIC B

ID—patient number; ESBL—extended-spectrum beta-lactamase; CR—carbapenem-resistant; VRE—vancomycin-resistant enterococcus; ** isolate demonstrates multidrug-resistance phenotype, but resistance genes were not detected.

**Table 3:** Primers and PCR conditions.

Target	Primer Sequence	T	Products Size	Reference
TEM	F: ATA AAA TTC TTG AAG AC R: TTA CCA ATG CTT AAT CA	43 °C	1075 bp	[57]
SHV	F: ACT GAA TGC GGC GCT TCC R: TCC CGC AGA TAA ATC A	61 °C	297 bp	[57]
CTX-M	F: CVA TGT GCA GYA CCA GTA A R: ARG TSA CCA GAA YMA GCG G	61 °C	585 bp	[57]
VIM	F: GAT GGT GTT TGG TCG CAT A R: CGA ATG CGC AGC ACC AG	54 °C	390 bp	[58]
IMP	F: GGA ATA GAG TGG CTT AAY TCT C R: GGT TTA AYA AAA CAA CCA CC	54 °C	270 bp	[58]
KPC	F: CGT CTA GTT CTG CTG TCT TG R: CTT GTC ATC CTT GTT AGG CG	57 °C	798 bp	[58]
NDM	F: GGT TTG GCG ATC TGG TTT TC R: CGG ATT GGC TCA TCA CGA TC	57 °C	621 bp	[58]
OXA-48	F: GCG TGG TTA AGG ATG AAC AC R: CAT CAA GTT CAA CCC AAC CG	57 °C	438 bp	[58]
ERIC	ERIC 1R: ATG TAA GCT CCT GGG GAT TCA C ERIC 2: AAG TAA GTG ACT GGG GTG AGC	54 °C	-	[62]
vanA/D	F: GAR GAY GGM WSC ATM CAR GGY R: MGT RAA WCC NGG CAK RGT RTT	51.3 °C	630 bp	[59]
vanA	F: CAT GAA TAG AAT AAA AGT TGC AAT A R: CCC CTT TAA CGC TAA TAC GAT CAA	54 °C	1030 bp	[60]
vanB	F: AAG CTA TGC AAG AAG CCA TG R: CCG ACA ATC AAA TCA TCC TC	54 °C	536 bp	[60]
RAPD	AB106: TGC TCT GCC C	32 °C	-	[63]

T—annealing temperature.

## Data Availability

Data are contained within the article.

## References

[1] Henig I., Zuckerman T. (2014). Hematopoietic stem cell transplantation-50 years of evolution and future perspectives. Rambam Maimonides Med. J..

[2] Worldwide Network for Blood & Marrow Transplantation. https://www.wbmt.org/wp-content/uploads/2023/07/2022-WBMT-Progress-Report.pdf.

[3] Jaing T.H. (2011). Complications of haematopoietic stem cell transplantation. ISBT Sci. Ser..

[4] Cao W., Guan L., Li X., Zhang R., Li L., Zhang S., Wang C., Xie X., Jiang Z., Wan D. (2021). Clinical Analysis of Bloodstream Infections During Agranulocytosis After Allogeneic Hematopoietic Stem Cell Transplantation. Infect. Drug Resist..

[5] Girmenia C., Bertaina A., Piciocchi A., Perruccio K., Algarotti A., Busca A., Cattaneo C., Raiola A.M., Guidi S., Iori A.P. (2017). Gruppo Italiano Trapianto di Midollo Osseo (GITMO) and Associazione Microbiologi Clinici Italiani (AMCLI). Incidence, Risk Factors and Outcome of Pre-engraftment Gram-Negative Bacteremia After Allogeneic and Autologous Hematopoietic Stem Cell Transplantation: An Italian Prospective Multicenter Survey. Clin. Infect. Dis..

[6] Ferreira A.M., Moreira F., Guimaraes T., Spadão F., Ramos J.F., Batista M.V., Filho J.S., Costa S.F., Rocha V. (2018). Epidemiology, risk factors and outcomes of multi-drug-resistant bloodstream infections in haematopoietic stem cell transplant recipients: Importance of previous gut colonization. J. Hosp. Infect..

[7] Kelly M.S., Ward D.V., Severyn C.J., Arshad M., Heston S.M., Jenkins K., Martin P.L., McGill L., Stokhuyzen A., Bhattarai S.K. (2019). Gut Colonization Preceding Mucosal Barrier Injury Bloodstream Infection in Pediatric Hematopoietic Stem Cell Transplantation Recipients. Biol. Blood Marrow Transplant..

[8] Alrstom A., Alsuliman T., Daher N., Abouharb R. (2021). The Impact of Modifying Empirical Antibiotic Therapy Based on Intestinal Colonization Status on Clinical Outcomes of Febrile Neutropenic Patients. Infect. Chemother..

[9] Taur Y., Xavier J.B., Lipuma L., Ubeda C., Goldberg J., Gobourne A., Lee Y.J., Dubin K.A., Socci N.D., Viale A. (2012). Intestinal domination and the risk of bacteremia in patients undergoing allogeneic hematopoietic stem cell transplantation. Clin. Infect. Dis..

[10] Schinas G., Skintzi K., De Lastic A.L., Rodi M., Gogos C., Mouzaki A., Akinosoglou K. (2023). Patterns, Cost, and Immunological Response of MDR vs. Non MDR-Bacteremia: A Prospective Cohort Study. Pathogens.

[11] Krawiec K.M., Strzałka P., Stańczak K., Czemerska M., Szmigielska A., Grzybowska-Izydorczyk O., Wierzbowska A., Pluta A. (2024). Evaluation of colonization and infection profile in allogeneic hematopoietic stem cell transplantation recipients. Acta Haematol. Pol..

[12] Perez P., Patiño J., Estacio M., Pino J., Manzi E., Medina D. (2020). Bacteremia in pediatric patients with hematopoietic stem cell transplantation. Hematol. Transfus. Cell Ther..

[13] Cao W., Zhang J., Bian Z., Li L., Zhang S., Qin Y., Wan D., Jiang Z., Zhang R. (2022). Active Screening of Intestinal Colonization of Carbapenem-Resistant Enterobacteriaceae for Subsequent Bloodstream Infection in Allogeneic Hematopoietic Stem Cell Transplantation. Infect. Drug Resist..

[14] Niyazi D., Micheva I., Dokova K., Stoeva T. (2023). Incidence, Risk Factors and Outcome of Bloodstream Infections in Patients After Hematopoietic Stem-Cell Transplantation: A Single Center Study. Indian J. Hematol. Blood Transfus..

[15] Scheich S., Lindner S., Koenig R., Reinheimer C., Wichelhaus T.A., Hogardt M., Besier S., Kempf V.A.J., Kessel J., Martin H. (2018). Clinical impact of colonization with multidrug-resistant organisms on outcome after allogeneic stem cell transplantation in patients with acute myeloid leukemia. Cancer.

[16] Bilinski J., Robak K., Peric Z., Marchel H., Karakulska-Prystupiuk E., Halaburda K., Rusicka P., Swoboda-Kopec E., Wroblewska M., Wiktor-Jedrzejczak W. (2016). Impact of Gut Colonization by Antibiotic-Resistant Bacteria on the Outcomes of Allogeneic Hematopoietic Stem Cell Transplantation: A Retrospective, Single-Center Study. Biol. Blood Marrow Transplant..

[17] Ford C.D., Gazdik M.A., Lopansri B.K., Webb B., Mitchell B., Coombs J., Hoda D., Petersen F.B. (2017). Vancomycin-Resistant Enterococcus Colonization and Bacteremia and Hematopoietic Stem Cell Transplantation Outcomes. Biol. Blood Marrow Transplant..

[18] Scheich S., Reinheimer C., Brandt C., Wichelhaus T.A., Hogardt M., Kempf V.A.J., Brunnberg U., Brandts C., Ballo O., von Metzler I. (2017). Clinical Impact of Colonization with Multidrug-Resistant Organisms on Outcome after Autologous Stem Cell Transplantation: A Retrospective Single-Center Study. Biol. Blood Marrow Transplant..

[19] Giannella M., Bartoletti M., Campoli C., Rinaldi M., Coladonato S., Pascale R., Tedeschi S., Ambretti S., Cristini F., Tumietto F. (2019). The impact of carbapenemase-producing Enterobacteriaceae colonization on infection risk after liver transplantation: A prospective observational cohort study. Clin. Microbiol. Infect..

[20] Le Bastard Q., Chevallier P., Montassier E. (2021). Gut microbiome in allogeneic hematopoietic stem cell transplantation and specific changes associated with acute graft vs host disease. World J. Gastroenterol..

[21] Arias Ramos D., Hoyos Pulgarín J.A., Moreno Gómez G.A., Alzate J.A., Olaya Gómez J.C., Cortés Bonilla I., Vargas Mosquera C. (2020). Geographic mapping of Enterobacteriaceae with extended-spectrum β-lactamase (ESBL) phenotype in Pereira, Colombia. BMC Infect. Dis..

[22] Kurittu P., Khakipoor B., Jalava J., Karhukorpi J., Heikinheimo A. (2022). Whole-Genome Sequencing of Extended-Spectrum Beta-Lactamase-Producing Escherichia coli From Human Infections in Finland Revealed Isolates Belonging to Internationally Successful ST131-C1-M27 Subclade but Distinct from Non-human Sources. Front. Microbiol..

[23] Wang Z., Lu Q., Mao X., Li L., Dou J., He Q., Shao H., Luo Q. (2022). Prevalence of Extended-Spectrum β-Lactamase-Resistant Genes in Escherichia coli Isolates from Central China during 2016–2019. Animals.

[24] Satlin M.J., Chavda K.D., Baker T.M., Chen L., Shashkina E., Soave R., Small C.B., Jacobs S.E., Shore T.B., van Besien K. (2018). Colonization with Levofloxacin-resistant Extended-spectrum β-Lactamase-producing Enterobacteriaceae and Risk of Bacteremia in Hematopoietic Stem Cell Transplant Recipients. Clin. Infect. Dis..

[25] Harris A.D., McGregor J.C., Johnson J.A., Strauss S.M., Moore A.C., Standiford H.C., Hebden J.N., Morris J.G. (2007). Risk factors for colonization with extended-spectrum beta-lactamase-producing bacteria and intensive care unit admission. Emerg. Infect. Dis..

[26] Schneider I., Keuleyan E., Rasshofer R., Markovska R., Queenan A.M., Bauernfeind A. (2008). VIM-15 and VIM-16, two new VIM-2-like metallo-beta-lactamases in Pseudomonas aeruginosa isolates from Bulgaria and Germany. Antimicrob. Agents Chemother..

[27] Strateva T., Setchanova L., Peykov S. (2021). Characterization of a Bulgarian VIM-2 metallo-β-lactamase-producing Pseudomonas aeruginosa clinical isolate belonging to the high-risk sequence type 111. Infect. Dis..

[28] Falcone M., Mezzatesta M.L., Perilli M., Forcella C., Giordano A., Cafiso V., Amicosante G., Stefani S., Venditti M. (2009). Infections with VIM-1 metallo-{beta}-lactamase-producing enterobacter cloacae and their correlation with clinical outcome. J. Clin. Microbiol..

[29] Heller I., Grif K., Orth D. (2012). Emergence of VIM-1-carbapenemase-producing Enterobacter cloacae in Tyrol, Austria. J. Med. Microbiol..

[30] Luo H., Chen X., Jiang Z., Yan Q. (2024). Prevalence of and risk factors for intestinal colonisation by multidrug-resistant Gram-negative bacteria in patients with haematological malignancies: A systematic review and meta-analysis. Int. J. Antimicrob. Agents.

[31] Stankova P., Markovska R., Boyanova L., Mitov I. (2020). Antibiotic susceptibility of intestinal isolates suspected of esbl/carbapenemase production of the order of esbl/carbapenemase production of the order enterobacterales enterobacterales isolated from hospitalized patients isolated from hospitalized patients and healthy individuals for the period 2017–2018. Mod. Med..

[32] Sahitya D.S.K., Jandiyal A., Jain A., Senapati J., Nanda S., Aggarwal M., Kumar P., Mohapatra S., Ray P., Malhotra P. (2021). Prevention and management of carbapenem-resistant Enterobacteriaceae in haematopoietic cell transplantation. Ther. Adv. Infect. Dis..

[33] Kömürcü B., Tigen E.T., Toptaş T., Tuğlular F.T., Korten V. (2020). Rectal colonization with multidrug-resistant gram-negative bacteria in patients with hematological malignancies: A prospective study. Expert. Rev. Hematol..

[34] Baidya A., Kodan P., Fazal F., Tsering S., Menon P.R., Jorwal P., Chowdhury U.K. (2019). Stenotrophomonas maltophilia: More than Just a Colonizer!. Indian J. Crit. Care Med..

[35] Garrison M.W., Anderson D.E., Campbell D.M., Carroll K.C., Malone C.L., Anderson J.D., Hollis R.J., Pfaller M.A. (1996). Stenotrophomonas maltophilia: Emergence of multidrug-resistant strains during therapy and in an in vitro pharmacodynamic chamber model. Antimicrob. Agents Chemother..

[36] Raad M., Abou Haidar M., Ibrahim R., Rahal R., Abou Jaoude J., Harmouche C., Habr B., Ayoub E., Saliba G., Sleilaty G. (2023). Stenotrophomonas maltophilia pneumonia in critical COVID-19 patients. Sci. Rep..

[37] Hitkova H.Y., Hristova P.M. (2019). Enterococcus and enterococcus-like organisms recovered in faecal screening for vancomycin-resistance. J. IMAB.

[38] Strateva T., Sirakov I., Dimov S., Trifonova A., Savov E., Mitov I. (2018). Clonal spread of vanA Enterococcus faecium sequence type 203 in Bulgarian hospitals. Infect. Dis..

[39] Mullié C., Lemonnier D., Adjidé C.C., Maizel J., Mismacque G., Cappe A., Carles T., Pierson-Marchandise M., Zerbib Y. (2022). Nosocomial outbreak of monoclonal VIM carbapenemase-producing Enterobacter cloacae complex in an intensive care unit during the COVID-19 pandemic: An integrated approach. J. Hosp. Infect..

[40] European Centre for Disease Prevention and Control. https://www.ecdc.europa.eu/en/publications-data/antimicrobial-resistance-eueea-ears-net-annual-epidemiological-report-2020.

[41] Kharrat M., Chebbi Y., Ben Tanfous F., Lakhal A., Ladeb S., Othmen T.B., Achour W. (2018). Extended spectrum beta-lactamase-producing Enterobacteriaceae infections in hematopoietic stem cell transplant recipients: Epidemiology and molecular characterization. Int. J. Antimicrob. Agents.

[42] Uemura M., Imataki O., Uchida S., Nakayama-Imaohji H., Ohue Y., Matsuka H., Mori H., Dobashi H., Kuwahara T., Kadowaki N. (2017). Strain-specific transmission in an outbreak of ESBL-producing Enterobacteriaceae in the hemato-oncology care unit: A cohort study. BMC Infect. Dis..

[43] Markovska R., Stankova P., Stoeva T., Murdjeva M., Marteva-Proevska Y., Ivanova D., Sredkova M., Petrova A., Mihova K., Boyanova L. (2022). Dissemination of High-Risk Clones Enterobacterales among Bulgarian Fecal Carriage Isolates. Microorganisms.

[44] Ramirez D., Giron M. (2024). Enterobacter Infections. [Updated 2023 Jun 26]. StatPearls.

[45] Sanders W.E., Sanders C.C. (1997). *Enterobacter* spp.: Pathogens poised to flourish at the turn of the century. Clin. Microbiol. Rev..

[46] Lee C.C., Lee N.Y., Yan J.J., Lee H.C., Chen P.L., Chang C.M., Wu C.J., Ko N.Y., Wang L.R., Chi C.H. (2010). Bacteremia due to extended-spectrum-beta-lactamase-producing Enterobacter cloacae: Role of carbapenem therapy. Antimicrob. Agents Chemother..

[47] Dimitrova D., Stoeva T., Markovska R., Stankova P., Mihova K., Kaneva R., Mitov I. (2019). Molecular Epidemiology of Multidrug Resistant Enterobacter cloacae blood isolates from a University Hospital. J. IMAB.

[48] Sid Ahmed M.A., Hamid J.M., Hassan A.M.M., Abu Jarir S., Bashir Ibrahim E., Abdel Hadi H. (2024). Phenotypic and Genotypic Characterization of Pan-Drug-Resistant Klebsiella pneumoniae Isolated in Qatar. Antibiotics.

[49] Sleiman A., Awada B., Mocadie M., Sherri N., Haraoui L.P., Baby V., Araj G.F., Kanj S.S., Rizk N., Matar G.M. (2021). An unequivocal superbug: PDR Klebsiella pneumoniae with an arsenal of resistance and virulence factor genes. J. Infect. Dev. Ctries..

[50] Fatima S., Liaqat F., Akbar A., Sahfee M., Samad A., Anwar M., Iqbal S., Khan S.A., Sadia H., Makai G. (2021). Virulent and multidrug-resistant Klebsiella pneumoniae from clinical samples in Balochistan. Int. Wound J..

[51] Weterings V., van Oosten A., Nieuwkoop E., Nelson J., Voss A., Wintermans B., van Lieshout J., Kluytmans J., Veenemans J. (2021). Management of a hospital-wide vancomycin-resistant Enterococcus faecium outbreak in a Dutch general hospital, 2014-2017: Successful control using a restrictive screening strategy. Antimicrob. Resist. Infect. Control.

[52] Jabbari Shiadeh S.M., Pormohammad A., Hashemi A., Lak P. (2019). Global prevalence of antibiotic resistance in blood-isolated Enterococcus faecalis and Enterococcus faecium: A systematic review and meta-analysis. Infect. Drug Resist..

[53] Rangberg A., Larsen A.L., Kacelnik O., Sæther H.S., Bjørland M., Ringstad J., Jonassen C.M. (2019). Molecular analysis and epidemiological typing of Vancomycin-resistant Enterococcus outbreak strains. Sci. Rep..

[54] García Martínez de Artola D., Castro B., Ramos M.J., Díaz Cuevas Z., Lakhwani S., Lecuona M. (2017). Outbreak of vancomycin-resistant enterococcus on a haematology ward: Management and control. J. Infect. Prev..

[55] Hughes A., Ballard S., Sullivan S., Marshall C. (2019). An outbreak of vanA vancomycin-resistant Enterococcus faecium in a hospital with endemic vanB VRE. Infect. Dis. Health.

[56] European Committee on Antimicrobial Susceptibility Testing. https://www.eucast.org/ast_of_bacteria/previous_versions_of_documents.

[57] Markovska R., Schneider I., Keuleyan E., Sredkova M., Ivanova D., Markova B., Lazarova G., Dragijeva E., Savov E., Haydouchka I. (2008). Extended-spectrum beta-lactamase-producing Enterobacteriaceae in Bulgarian hospitals. Microb. Drug Resist..

[58] Poirel L., Walsh T.R., Cuvillier V., Nordmann P. (2011). Multiplex PCR for detection of acquired carbapenemase genes. Diagn. Microbiol. Infect. Dis..

[59] Strateva T., Atanasova D., Mitov I., Sirakov I., Katrandjieva A. (2014). Emergence of VanB phenotype-vanA genotype Enterococcus faecium clinical isolate in Bulgaria. Braz. J. Infect. Dis..

[60] Kariyama R., Mitsuhata R., Chow J.W., Clewell D.B., Kumon H. (2000). Simple and reliable multiplex PCR assay for surveillance isolates of vancomycin-resistant enterococci. J. Clin. Microbiol..

[61] Lévesque C., Piché L., Larose C., Roy P.H. (1995). PCR mapping of integrons reveals several novel combinations of resistance genes. Antimicrob. Agents Chemother..

[62] Bakhshi B., Afshari N., Fallah F. (2018). Enterobacterial repetitive intergenic consensus (ERIC)-PCR analysis as a reliable evidence for suspected *Shigella* spp. outbreaks. Braz. J. Microbiol..

[63] Niyazi D.S. (2022). Investigation of Bacteraemia and Invasive Fungal Infections in Patients Following Autologous and Allogeneic Hematopoietic Stem Cell Transplantation. Ph.D. Thesis.

